# Quantitative accuracy of ^177^Lu SPECT imaging for molecular radiotherapy

**DOI:** 10.1371/journal.pone.0182888

**Published:** 2017-08-14

**Authors:** Emilio Mezzenga, Vincenzo D’Errico, Marco D’Arienzo, Lidia Strigari, Koutla Panagiota, Federica Matteucci, Stefano Severi, Giovanni Paganelli, Andrew Fenwick, David Bianchini, Francesco Marcocci, Anna Sarnelli

**Affiliations:** 1 Medical Physics Unit, Istituto Scientifico Romagnolo per lo Studio e la Cura dei Tumori (IRST) IRCCS, Meldola, Italy; 2 National Institute of Ionizing Radiation Metrology, ENEA CR Casaccia, Rome, Italy; 3 Laboratory of Medical Physics and Expert Systems, Regina Elena National Cancer Institute, Rome, Italy; 4 Department of Physics, Aristotle University of Thessaloniki, Thessaloniki, Greece; 5 Nuclear Medicine Unit, Istituto Scientifico Romagnolo per lo Studio e la Cura dei Tumori (IRST) IRCCS, Meldola, Italy; 6 National Physical Laboratory, Hampton Road, Teddington, United Kingdom; University of Chicago, UNITED STATES

## Abstract

The purpose of this study is to investigate the optimal reference geometry for gamma camera calibration. Yet another question of interest was to assess the influence of the number of 3D Ordered Subsets Expectation Maximization (3D-OSEM) updates on activity quantification for SPECT imaging with ^177^Lu. The accuracy of ^177^Lu activity quantification was assessed both in small and in large objects. Two different reference geometries, namely a cylindrical homogeneous phantom and a Jaszczak 16 ml sphere surrounded by cold water, were used to determine the gamma camera calibration factor of a commercial SPECT/CT system. Moreover, the noise level and the concentration recovery coefficient were evaluated as a function of the number of 3D-OSEM updates by using the SPECT/CT images of the reference geometry phantoms and those of a cold Jaszczak phantom with three hot spheres (16ml, 8ml and 4ml), respectively. The optimal choice of the number of 3D-OSEM updates was based on a compromise between the noise level achievable in the reconstructed SPECT images and the concentration recovery coefficients. The quantitative accuracy achievable was finally validated on a test phantom, where a spherical insert composed of two concentric spheres was used to simulate a lesion in a warm background. Our data confirm and extend previous observations. Using the calibration factor obtained with the cylindrical homogeneous phantom and the Jaszczak 16 ml sphere, the recovered activity in the test phantom was underestimated by -16.4% and -24.8%, respectively. Our work has led us to conclude that gamma camera calibration performed with large homogeneous phantom outperforms calibration executed with the Jaszczak 16ml sphere. Furthermore, the results obtained support the assumption that approximately 50 OSEM updates represent a good trade-off to reach convergence in small volumes, meanwhile minimizing the noise level.

## Introduction

In recent years, molecular radiotherapy (MRT) based on peptide receptor radionuclide therapy has gained popularity for treatment of neuroendocrine tumors [1–4]. In particular, peptides labeled with Lutetium-177 (^177^Lu) have gained today an established use in the treatment of this disease [5–10]. In addition, encouraging results have also been obtained with theranostic radiopharmaceuticals in metastatic prostate cancer patients, indicating that the use and importance of ^177^Lu is expected to increase in the coming years also in the management of these patients [11–14]. Even if promising results are obtained in terms of treatment outcomes from patients treated with ^177^Lu using fixed activities, it is expected that personalized dose assessment could further improve the clinical outcomes in terms of the tumor control and reduction of the normal tissue effects.

From the physical point of view, ^177^Lu has several pros: (a) a concentrated energy deposition due to its low-energy beta emissions; (b) a favorable half-life; (c) a gamma emissions enabling imaging to evaluate the radiotracer biodistribution. A preliminary mandatory step to perform personalized dosimetry relies on the possibility of having accurate quantitative information from reconstructed images [15,16]. In particular, SPECT/CT systems have the potential of enabling the conversion from counts in each voxel into activity values.

The general recommendations outlined on the Medical Internal Radiation Dose (MIRD) pamphlet No. 23 [17] and No. 26 [18], the latter specifically dedicated to ^177^Lu dosimetry, do not state uniquely defined procedures, protocols or correction factors. In addition, MIRD No. 26 highlighted that high variations in voxel counts may depend on the reconstruction process and not necessarily on the heterogeneous biologic uptake of the radiopharmaceutical [18]. Along these lines, MIRD No. 23 states that a larger or lower number of iterations should be used if the mean absorbed dose or the dose volume histograms, respectively, are to be calculated [17]. Consequently, different calibration geometries, data acquisition and processing methods have been adopted for obtaining the calibration of SPECT/CT scanner [19–23].

Beauregard et al. [19] validated a quantitative SPECT method using a commercially available SPECT/CT system and its software supporting attenuation and scatter correction factors. De Nijs et al. [20] investigated the conversion factors from counts to activity focusing on the collimator type, the energy windows and the scatter correction techniques. Sanders et al. [21] highlighted that the calibration factor calculated for each set of reconstruction parameters (i.e. number of iterations and subsets) was sensitive only to the choice of photopeak (113 keV vs 208 keV). Scherbinin et al. [22] examined the ability of different methods including attenuation and scatter correction, resolution loss and contamination in order to accurately reconstruct the distributions of ^177^Lu activity. Hippeläinen et al. [23] found higher concentration recovery coefficients when the attenuation, collimator-detector response and scatter correction were applied to the reconstruction of images of an anthropomorphic phantom. Finally, Zeintl et al. [24] used quasi-analytic simulation of cross-calibrated clinical SPECT/CT to determine the correction factors to be applied to reconstructed images.

In this context, our work focuses on the investigation of two different reference geometries for SPECT calibration and on the image reconstruction process in order to evaluate the percent deviation in small volumes for dosimetry purposes. To the best of our knowledge, the relation between the noise level and strategy of reconstruction in the accuracy of quantitative SPECT imaging using ^177^Lu has never been investigated in such a complex geometry.

For the sake of consistency, the present work has been organized into three sections:

1. the first part deals with the implementation of two different reference geometries (easy to be implemented in the clinical practice) for SPECT calibration, considering different image reconstruction parameters (i.e. number of 3D-OSEM updates);
2. the second part of the paper aims to establish the concentration recovery coefficients for spherical objects and study the impact of noise level as a function of the number of 3D-OSEM updates, using the calibration factors previously assessed. The optimal choice of the number of 3D-OSEM updates is based on a compromise between the noise level achievable in the reconstructed SPECT images and the concentration recovery coefficients;
3. finally, the quantification procedure analyzed in the previous steps was validated in anthropomorphic geometry, i.e. a torso phantom provided with a hot spherical shell mimicking a solid tumor surrounded by a fainter circular area background.

## Materials and methods

### Reference calibration geometries and recovery coefficient phantom

**Fig 1:**
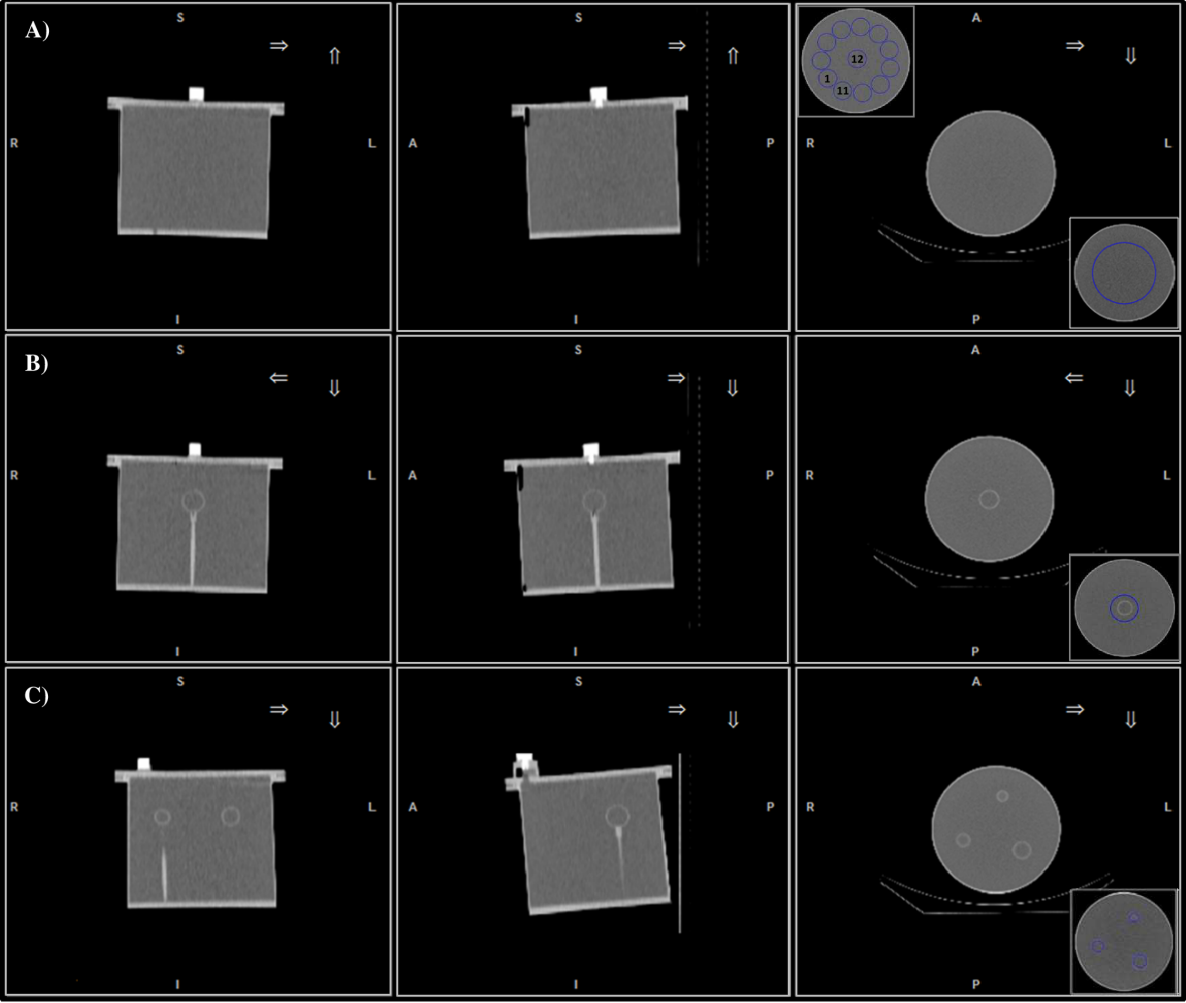
CT images of the phantoms used for SPECT calibration and activity recovery evaluation. From left to right: coronal (first column), sagittal (second column) and transaxial (third column) CT views of A) Hp, B) Js and C) Rp phantom. The insets shown in the third column refer to the different volumes of interest (blue color) used for SPECT calibration (right inferior corner of A) and B)), noise (left superior corner of A), together with the location of VOI_w_) and activity recovery (right inferior corner of C)).

Calibration of the SPECT system was performed by using two different reference geometries: (a) a 6.4 l cylindrical homogeneous phantom (Data Spectrum Corporation, Hillsborough, USA) filled with a ^177^Lu concentration of 0.11 MBq/ml; (b) a Jaszczak sphere of 16 ml in volume filled with a ^177^Lu concentration of 30.3 MBq/ml, fixed to the bottom of a cylindrical phantom (the same used in (a)) filled with cold water. Hereafter, the reference geometry (a) will be indicated as homogeneous phantom (Hp, Fig 1A), while the reference geometry (b) will be indicated as Jaszczak sphere phantom (Js, Fig 1B).

Finally, a third phantom (hereafter called Recovery phantom—Rp, Fig 1C) was used to assess the concentration recovery coefficients in spherical objects. The phantom is composed by three spheres of different volumes (16ml, 8ml and 4ml), each one filled with a ^177^Lu concentration of 1.13 MBq/ml and fixed to the bottom of a cylindrical phantom filled with cold water.

For these three phantoms, the accuracy of activity was assessed by means of the clinically available dose calibrator with an uncertainty of about 5%, and the activity concentration was referred to the time of SPECT acquisition.

The SPECT dead time was assessed using the dual source method, according to [25,26], and dead time corrections were evaluated for Hp and Js and applied to the reconstructed images.

### Validation geometry

**Fig 2:**
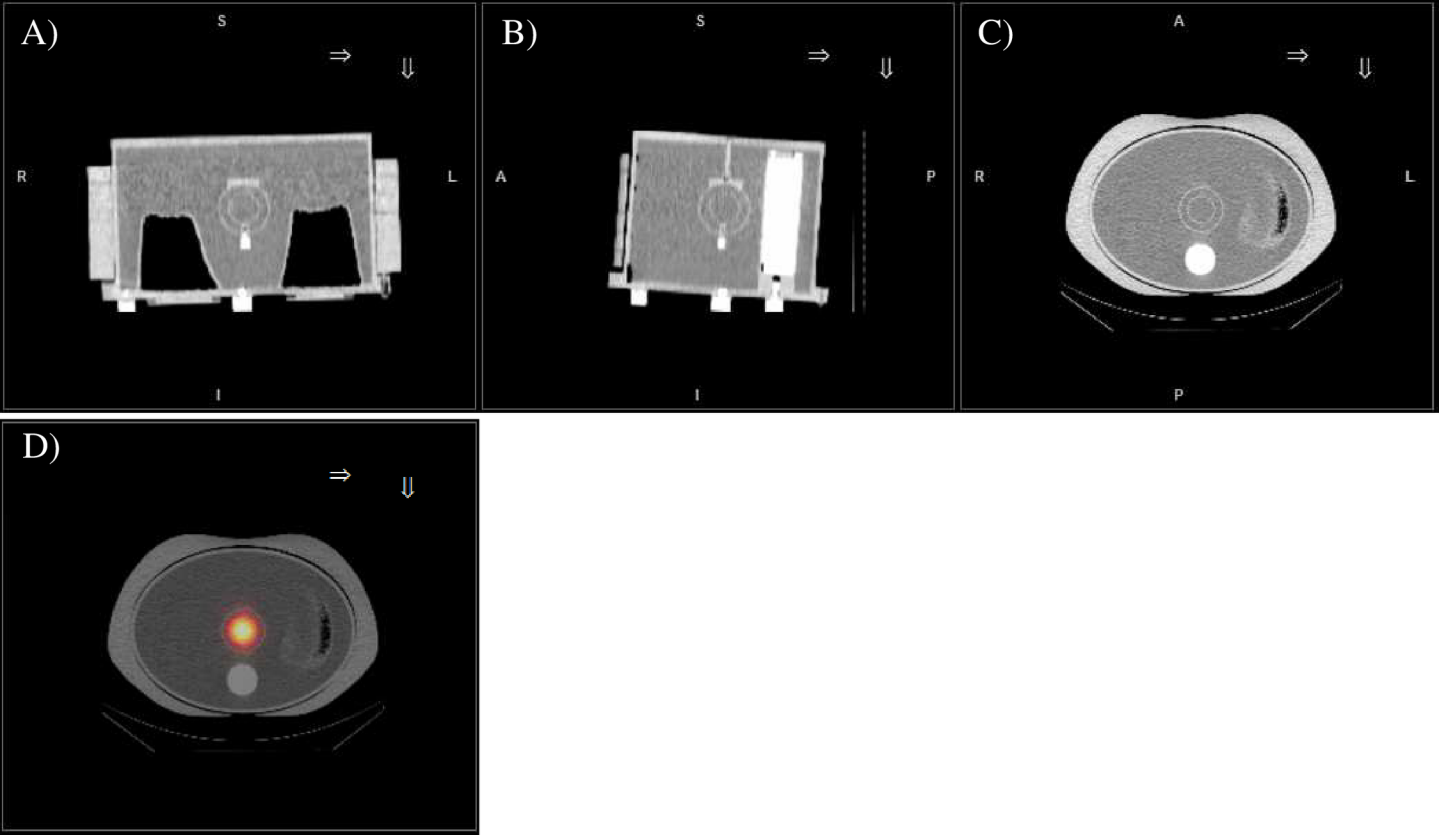
Validation anthropomorphic phantom. A) Coronal, B) sagittal and C) transaxial CT views of the validation anthropomorphic phantom. D) transaxial view of fused SPECT/CT images.

In order to assess the quantitative accuracy of our procedure for recovering the activity of small volume objects mimicking tumor lesions (i.e. high uptake region surrounded by a fainter region), a validation anthropomorphic phantom was used (Fig 2).

Specifically, we used an elliptical Jaszczak phantom representative of realistic clinical conditions. The phantom was provided with lungs, body contour rings, spine compartment and a spherical insert (representative of a tumor lesion) made of two concentric spheres. The spine insert was filled with bone equivalent solution of dipotassium hydrogen orthophosphate (K_2_HPO_4_) and the lungs were filled with lung equivalent material. In between the two lungs, the spherical insert composed of two concentric spheres of different volumes was placed and attached to the base plate of the phantom with the purpose to simulate a solid tumor (internal sphere, 18.3 mm diameter) with a fainter background region (external shell, 27.8 mm diameter). The inner sphere and the spherical shell were filled with an activity concentration of 2.7 MBq/ml and 0.2 MBq/ml, respectively. The remaining volume of the phantom was filled with cold water.

### SPECT/CT acquisition protocol

All acquisitions were performed using a hybrid SPECT/CT system (Discovery NM/CT 670, GE Healthcare, Milwaukee, USA), equipped with two gamma detector heads (9.5 mm NaI(Tl) crystal thickness of 40 cm axial by 54 cm diameter field of view), and an integrated CT component identical to a 16-slice-CT used in diagnostic CT imaging (model: Bright Speed 16, GE Healthcare, Milwaukee, USA). SPECT acquisitions were performed using the following parameters: 120 projections with 180° mode detector head, 30 seconds per projection, non-circular step-and-shot acquisition orbit, 128×128 matrix and 4.42×4.42 mm pixel size. For scatter correction, projection data were acquired in three energy windows using a parallel-hole medium energy general purpose (MEGP) collimator: a symmetrical 20% wide energy window was centered at 208 keV ^177^Lu photopeak (energy window:187.2 keV-228.8 keV), together with two 8.7% and 11.8% wide adjacent scatter windows, providing the upper and lower scatter windows, respectively. The SPECT acquisition was followed by a CT scan (120 kV, 80mAs, 1.375 pitch, 16x1.25 mm collimation, 3.75 slice thickness reconstruction). CT images were reconstructed with filtered back projection algorithm using the default convolution kernel for routine low dose CT (LD-CT) examination of abdomen.

### Image reconstructions

SPECT/CT data processing was carried out on a dedicated workstation (Xeleris 3.1108, GE Healthcare, Milwaukee, USA), provided with a software from the same vendor (Dosimetry Toolkit Package, GE Healthcare, Milwaukee, USA). It reconstructs the SPECT images by means of 3D-OSEM algorithm [27] including resolution recovery, scatter correction and attenuation correction. This last correction was performed by linear attenuation coefficient (μ) maps estimated from the acquired LD-CT. Reconstruction of SPECT images was performed by considering 5, 10, 15, 20 and 30 subsets, with a number of iterations from 1 to 7 with an incremental step of 1, and from 10 to 50 with a incremental step of 5. A total of 80 combinations have been considered for SPECT images related to the reference geometries. No pre- and post-reconstruction filters were used, and, at the end of the reconstruction process, the software re-bins the CT matrix to the SPECT one giving a 256×256 matrix dataset with a 2.21 mm isotropic voxel.

### Data analysis

All reconstructed SPECT images have been processed with MATLAB (The Mathworks, Inc., MA, US). For Hp, a cylindrical volume of interest (VOI) of about 2.0 l was used to calculate the calibration factor. To minimize the edge effects the VOI was designed with a minimum distance of 3 cm set from the inner boundaries of the phantom (Fig 1A, inset at the bottom right corner). For Js, the CT-based contour of the 16 ml sphere was isotropically expanded and a spherical VOI of 6 cm diameter was used for the calculation of the calibration factor (Fig 1B, inset at the bottom right corner). The total counts inside the VOIs were recorded for each combination of subset (*s*) and iteration (*i*) (hereafter the product *s* x *i* will be referred as equivalent iterations—*EI*). The SPECT calibration factor (or sensitivity—*S*), expressed in units of counts-per-second/MBq (cps/MBq) was calculated using Eq (1) [15]:

$$S_{j}(EI)=\frac{\frac{R_{j}(EI)}{V_{{VOI}_{j}}}}{c_{j}}\;\left\{ \begin{array}{c} j=1\;for\;Hp\\ j=2\;for\;Js \end{array}\right.$$ (1)

where *V*_*VOIj*_ is the volume of the considered VOI, *c*_*j*_ the activity concentration inside the phantom volume, and *R*_*j*_*(EI)* the decay-corrected counting rate [24] function of the considered *EI*.

The image noise for both reference geometries was evaluated by means of the coefficient of variation (*COV*), defined as the ratio between the standard deviation (*s*) and the average (*M*) of voxel counts inside the VOIs considered [21,28]. In particular, for Hp twelve 16 ml spherical VOIs (left superior corner of Fig 1A) were drawn at a distance of 15 mm from the edge of the phantom, and for each VOI_w_ (*w* = 1, 2, …, 12, see inset in left superior corner of Fig 1A) the *COV* was evaluated as reported in Eq (2):

$${COV}_{w}(EI)\%=100\times \frac{s_{w}(EI)}{M_{w}(EI)}$$ (2)

The *COV* related to the Hp geometry was evaluated as the mean value of the *COV*_*w*_. A 16 ml spherical VOI was chosen for Hp in analogy with the *COV* evaluation performed for Js. In fact, in this last calibration geometry a spherical VOI based on the CT-contour of the 16 ml sphere was considered, and the *COV* was evaluated according to Eq (2) for *w* = 1.

To estimate the quantitative accuracy achievable using the two calibration factors, the reconstructed SPECT images of the Rp were used to determine the concentration recovery coefficient (*cRC*) of each spheres considered. The *cRC* was estimated according to Eq (3) [21]:

$${cRC}_{k}(EI)=\frac{\left(\frac{R_{k(EI)}}{V_{k}∙S_{j}(EI)}\right)}{c}\;\left\{ \begin{array}{c} \;k=16\;ml\\ \;k=8\;ml\\ \;k=4\;ml \end{array}\right.$$ (3)

where *R*_*k*_*(EI*) is the count rate inside the CT-based contours of *k*-th sphere (Fig 1C, bottom right corner), *S*_*j*_*(EI)* the calculated SPECT calibration factor related to the *j*-th reference geometry, *V*_*k*_ the VOI related to the *k*-th sphere, and *c* refers to the sphere’s concentration.

The validation of the quantification procedure was performed with the anthropomorphic phantom (Fig 2) and the inner spherical insert, mimicking tumor lesions, was considered as a bench test. The difference between the true injected activity (*A*_*T*_) and the reconstructed one (*A*_*R*_) was estimated according to Eq (4):

$$D_{j}=100*\frac{\left(A_{R,j}(EI)-A_{T}\right)}{A_{T}}\;\left\{ \begin{array}{c} j=1\;for\;Hp\\ j=2\;for\;Js \end{array}\right.$$ (4)

where *A*_*R*_*(EI)* was calculated from the totals counts inside the CT-based contours of the inner spherical insert, converted into activity by using *S* values (Eq 1) for the Hp and Js geometries.

## Results

**Fig 3:**
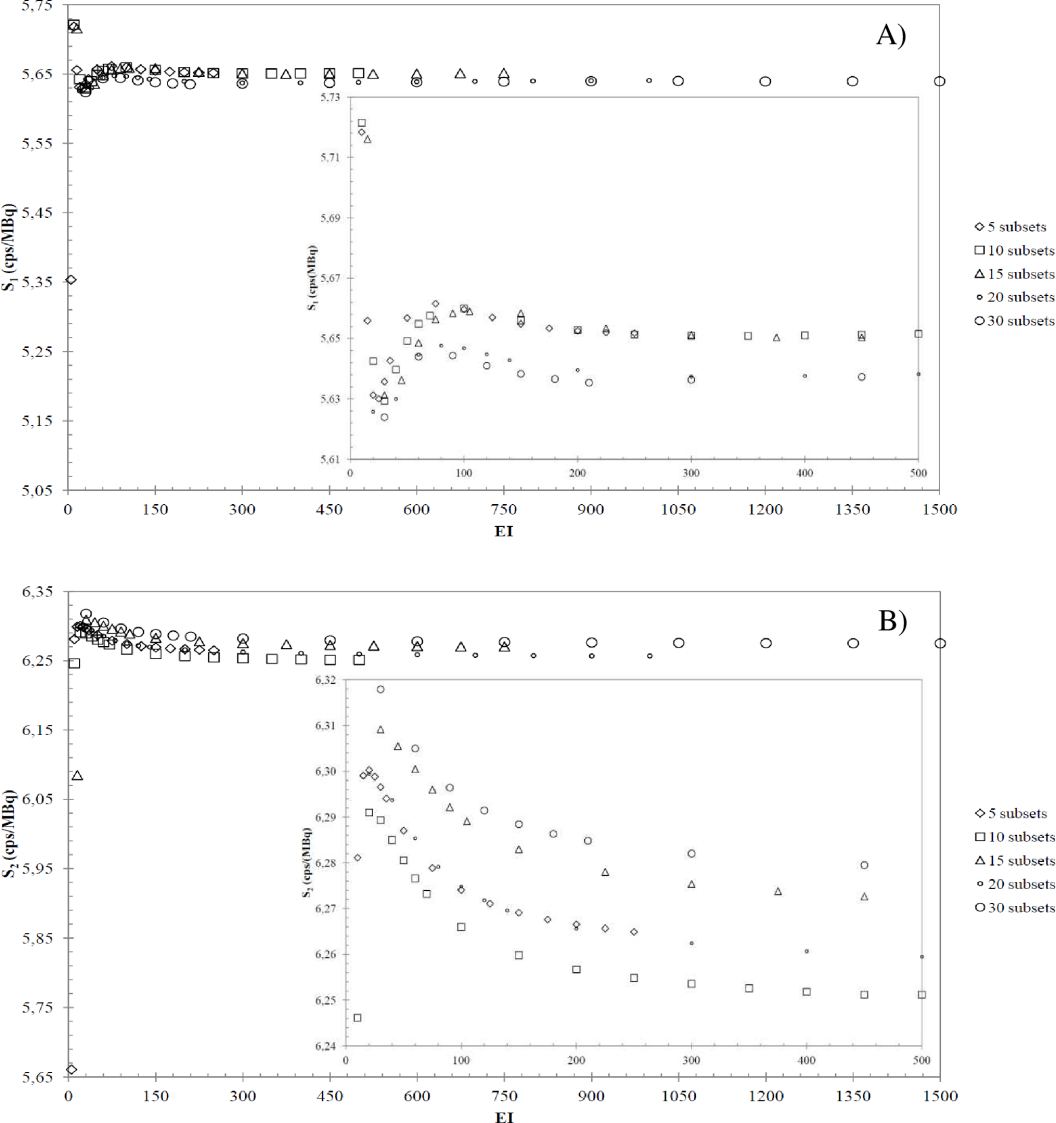
SPECT calibration factors versus *EI*. Calibration factors (*S*) as a function of *EI* for A) Hp and B) Js geometries, respectively. The insets reported are zoom views of the initial *S* trend versus *EI*.

Fig 3 shows the calibration factors (*S*) as a function of *EI* obtained for A) Hp and B) Js, respectively. Each plot refers to the number of subsets chosen, as reported in the legend. For completeness and clarity of the results, the insets highlight the trend of the dataplots for a restricted interval of *S* and *EI*. In case of Hp (Fig 3A), for all subsets the *S* values increase as a function of *EI* for *EI*<100, while for *EI*>100 the *S* values decreases as a function of *EI*, untill a plateau is reached. In the case of Js (Fig 3B), the *S* values decrease with increasing *EIs*, untill a plateau is reached for 15, 20 and 30 subsets, while for 5 and 10 subsets the data trend is similar to Hp one, but the S plateau is reached for EI>200.These different trends can be ascribed to the different volume of the Hp and Js geometries [18]. In fact, fixing the number of subsets, the EI required for convergence depend on the volume: hence, the plateaus of S start from different EI values for Hp and Js geometries.

**Fig 4:**
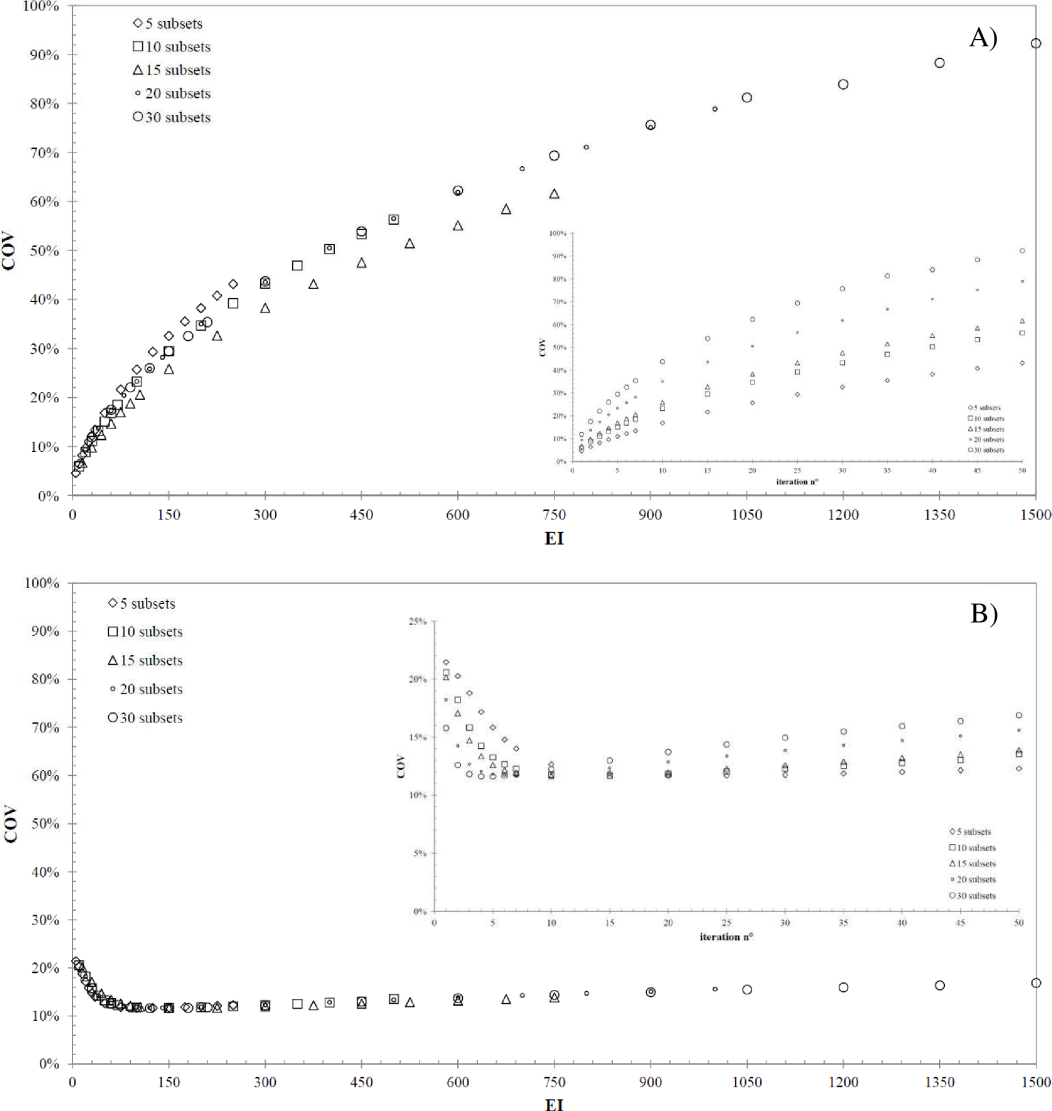
Relationship between *COV* and *EI*. *COV* as a function of *EI* and the subsets considered for A) Hp and B) Js geometry. The insets show the *COV* values as a function of the number of iterations.

Fig 4 shows the *COV* values as a function of *EI* for A) Hp and B) Js geometries, respectively, for all subsets of interest. In order to highlight the dependence between *COV* and the number of subsets and iterations, the *COV* data are reported as a function of the number of iterations in the insets of Fig 4 for A) Hp and B) Js geometries and in Table 1 and Table 2, respectively. The data trend shown in Fig 4A confirms the expected influence of 3D-OSEM algorithm on noise values in the SPECT images: fixing the number of subsets, the larger the number of iterations the larger the *COV*, and the same happens fixing the number of iterations (see also data in Table 1). Differently, in the case of Js (Fig 4B) and for all subsets, the *COV* reaches a minimum in the range between 50 and 150 *EI*, that is approximately constant and equals roughly to 12%. After this *EI* range, the Js *COV* increases with the same trend shown in Fig 4A. The information about the number of iterations at which the *COV* reaches its minimum can be derived for each number of subsets from the insets of Fig 4B and Table 2. The different *COV* trends observed for Hp and Js are the results of the 3D-OSEM algorithm applied to the different volumes. Furthermore, the Hp *COV* provides information about the noise level in large anatomical regions where the radiopharmaceutical is supposed to be uniformly distributed (i.e. tissues or organs with uniform uptake of the radionuclide), while the Js *COV* provides the same information for a small volume, such as a lesion’s volume inside the patient. As a general rule, the noise behavior in the phantom will be highly local due to the fact that convergence occurs at different rates at different points in the image. Therefore, we studied the dependence of *COV* as a function of VOI location inside the Hp phantom (left superior corner of Fig 1A), varying the number of *EI*. The standard deviation of the twelve *COV* values (estimated considering the *VOI*_*w*_) represents the error that affect the noise measurements when a single VOI is chosen. Fixing the number of subsets, the standard deviation increases as the number of iterations increases, ranging between 0.7% (at *EI* equals to 5, i.e. 5*s*x1*i*) and 9.8% (at *EI* equals to 1500, i.e. 30*s*x50*i*). Based on these results, it is reasonable to assume that the error affecting the *COV* estimation for Js geometry is comparable to that for Hp one. Moreover, these results support the hypothesis that noise becomes dominant with increasing *EIs*.

**Table 1:** Results of the noise values for the Hp reference geometry.

iterations	5-subsets	10-subsets	15-subsets	20-subsets	30-subsets
1	4.58%*	5.95%*	6.72%*	9.47%*	11.88%*
2	6.46%*	8.86% *	9.82%*	13.72%*	17.51%
3	8.21%*	11.15% *	12.37%*	17.33%	22.08%
4	9.67%*	13.20% *	14.69% *	20.48%	25.99%
5	10.99%*	15.10% *	17.01%	23.29%	29.44%
6	12.23% *	16.86% *	18.78%	25.83%	32.53%
7	13.42% *	18.51%	20.60%	28.18%	35.37%
10	16.87% *	23.24%	25.83%	34.99%	43.70%
15	21.63%	29.48%	32.63%	43.51%	53.90%
20	25.72%	34.69%	38.29%	50.51%	62.24%
25	29.32%	39.21%	43.18%	56.54%	69.38%
30	32.56%	43.24%	47.54%	61.88%	75.64%
35	35.51%	46.90%	51.50%	66.69%	81.22%
40	38.23%	50.26%	55.13%	71.09%	83.93%
45	40.77%	53.38%	58.50%	75.14%	88.32%
50	43.15%	56.30%	61.65%	78.89%	92.29%

∗Combinations of iterations and subsets with a noise level lower than 17%.

**Table 2:** Results of the noise values for the Js reference geometry.

iterations	5-subsets	10-subsets	15-subsets	20-subsets	30-subsets
1	21.45%	20.58%	20.16%	18.21%	15.77%
2	20.26%	18.22%	17.08%*	14.26%	12.60%
3	18.78%	15.82%*	14.72%	12.69%	11.81%
4	17.17%	14.24%	13.38%	12.07%	11.62%
5	15.83%	13.27%	12.62%	11.82%	11.61%
6	14.79%*	12.66%	12.18%	11.73%	11.69%
7	14.01%	12.28%	11.92%	11.72%	11.80%
10	12.66%	11.77%	11.64%	11.88%	12.22%
15	11.87%	11.67%	11.72%	12.35%	13.00%
20	11.68%	11.82%	11.97%	12.87%	13.73%
25	11.68%	12.03%	12.27%	13.37%	14.38%
30	11.75%	12.28%	12.58%	13.85%	14.96%
35	11.87%	12.53%	12.90%	14.30%	15.48%
40	12.00%	12.79%	13.21%	14.71%	15.95%
45	12.14%	13.05%	13.51%	15.10%	16.38%
50	12.29%	13.55%	13.91%	15.60%	16.88%

**Fig 5:**
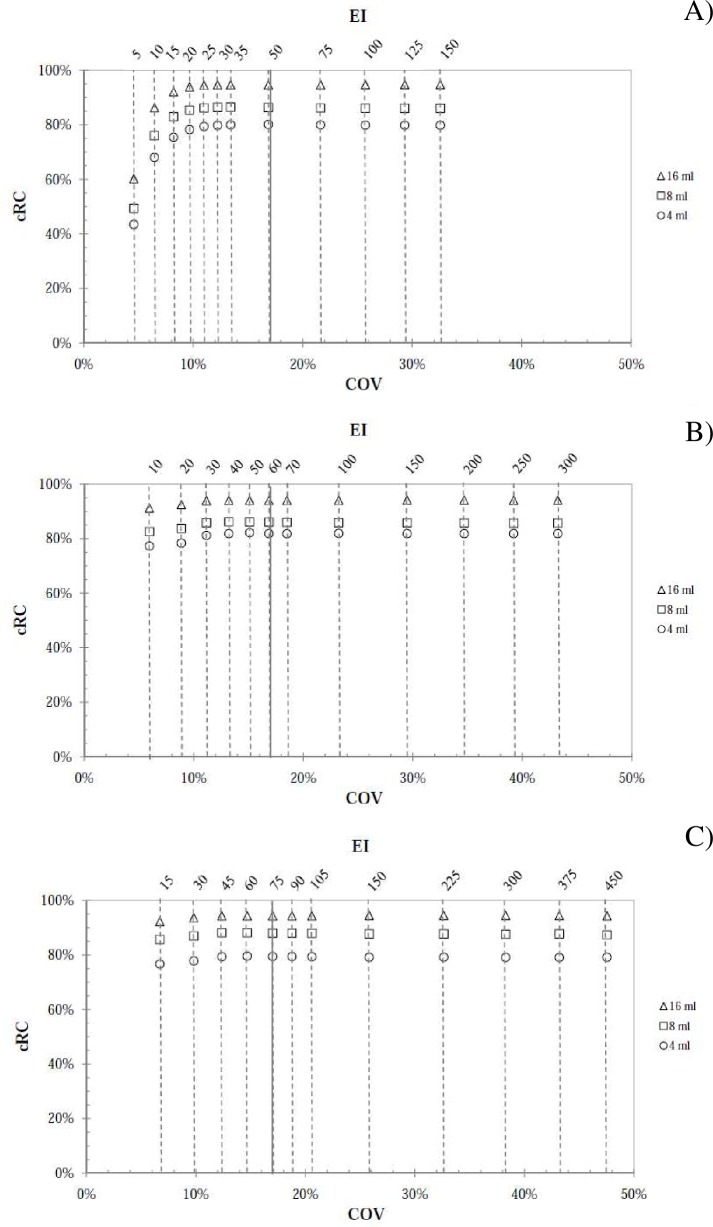
Concentration recovery coefficient for the Hp geometry. *cRCs* data versus *COV* and *EI* for the 16 ml, 8 ml and 4 ml spheres considered in the Rp phantom and using A) 5 subsets, B) 10 subsets and C) 15 subsets. The continuous vertical lines refer to the 17% noise threshold.

**Fig 6:**
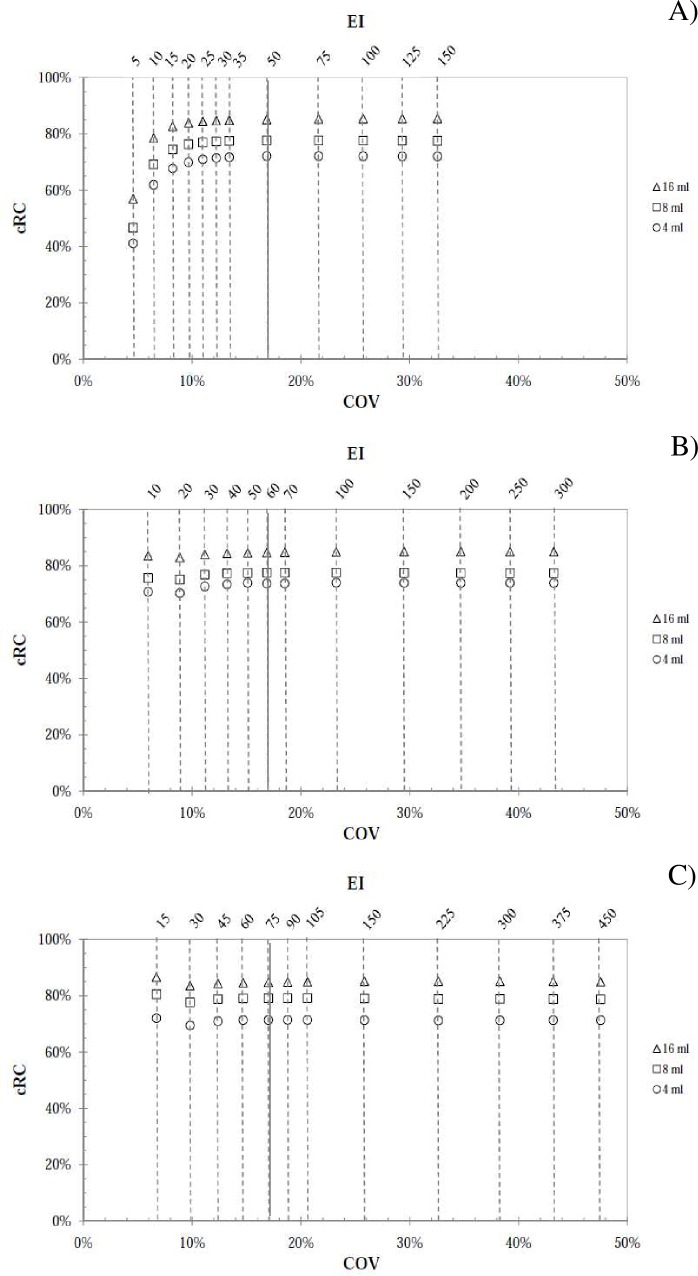
Concentration recovery coefficient for the the Js geometry. *cRCs* data versus *COV* and *EI* for the 16ml, 8ml and 4ml sphere considered in the Rp phantom and relative to A) 5 subsets, B) 10 subsets and C) 15 subsets. The continuous vertical lines refer to the 17% noise threshold.

∗Combinations of iterations and subsets for which the *cRC* plateaus start (Fig 5 and Fig 6).

In order to minimize the *COV* in the reconstructed images, the lowest *EI* values should be considered in the case of Hp geometry, as the Hp *COV* monotonically increases as a function of *EI*. In the case of the Js geometry, the *EI* at which the *COV* reaches its minimum should be considered, as a function of the subsets. In order to define a *COV* threshold suitable for both situations (large and small volumes), an *EI* value equal to 50 has been chosen because it corresponds to the lower bound of the *EI* range in which the *COV* related to the Js geometry is near to its minimum. For *EI* values equal to 50 the Hp *COV* ranges between 15% (i.e. 10*s*x5*i*) and 17% (i.e. 5*s*x10*i*) as shown in Fig 4A and Table 1. Conservatively, a *COV* value of 17% is assumed in the following as the *COV* threshold related to Hp geometry.

The *cRC* values were calculated for the spheres in the Rp phantom according to Eq (3) and the calibration factors (*S*) derived from the two calibration geometries. *cRC* and *COV* values are reported on the vertical and lower horizontal axes of Fig 5 (for Hp calibration factors) and Fig 6 (for Js calibration factors), respectively, with the corresponding *EIs* denoted on the upper horizontal axis. The plots in A), B) and C) (indicated at the top of the graph) refer only to 5, 10 and 15 subsets in both Fig 5 and Fig 6, while for the sake of brevity 20 and 30 subsets were not shown.

From a qualitative point of view, the trend of the data plot is the same for calibration factors from both reference geometries. Moreover, the *cRC* values in Fig 5 are larger than the ones in Fig 6, showing that the calibration factors from Hp are lower than that from Js for all *EIs* (Eq (3) and Fig 3). Considering a *cRC* value equal to 90% as a level of high reconstruction accuracy [17], the data plot of Fig 5 reach this level, while those reported in Fig 6 do not satisfy this requirement. In Table 3 are reported the data relative to Fig 5, referring to the 16 ml sphere.

**Table 3:** *cRC* values for the Rp phantom. The data in the table corresponds to the points in Fig 5 relative to the 16 ml sphere.

5-subsets		10-subsets		15-subsets	
EI	16 ml	EI	16 ml	EI	16 ml
5	60,20%	10	91,20%	15	92,20%
10	86,30%	20	92,50%	30	93,60%
15	92,10%	30	93,90%	45	94,30%
20	93,90%	40	94,10%	60	94,40%
25	94,50%	50	94,00%	75	94,40%
30	94,60%	60	94,00%	90	94,30%
35	94,60%	70	94,00%	105	94,40%
50	94,60%	100	94,00%	150	94,50%
75	94,60%	150	94,10%	225	94,50%
100	94,70%	200	94,10%	300	94,50%
125	94,70%	250	94,10%	375	94,50%
150	94,70%	300	94,10%	450	94,30%

As shown, the *cRC* values at fixed *EI* are very similar and the *cRC* convergence starts for *EI* greater than or equal to 30 for all reconstructions considered and for the 16 ml sphere. For the case of 20 and 30 subsets (plots not shown), the *cRC* data show the same trend but the convergence is reached at larger *EI*. Considering that for the Js geometry the minimum *COV* values lies in the *EI* range between 50 and 150, the *EI* equal to 50 (i.e. 10*s*x5*i*) can be chosen as our working point because is the best compromise between reducing the *COV* for both Hp and Js geometries (Table 1 and Table 2) and, at the same time, the *cRC* is in convergence. For this reason, the SPECT images of the validation phantom were reconstructed using *EI* equal to 50, and the percent difference between the injected and the reconstructed activity was evaluated for the inner spherical insert. The difference was D_1_ = -16.4% (Hp geometry) and D_2_ = -24.8% (Js geometry).

## Discussion

Nowadays, in patients with unresectable or metastatic neuroendocrine tumors molecular radiotherapy appears to be the most effective therapeutic strategy with limited side-effects [1]. For this reason, accurate quantitative imaging must be regarded as an essential integral part of the whole dosimetry procedure. A pivotal element in a patient-specific dosimetry approach using SPECT images is the accuracy of activity quantification inside tumor regions and organs at risk. This is strictly related to the calibration method used to convert SPECT data into activity, and to the SPECT reconstruction method used (i.e. correction for attenuation, scatter, collimator response, activity recovery and the number of 3D-OSEM algorithm updates). Our work focuses on the SPECT calibration method and, in particular, on the optimization of the number of 3D-OSEM updates when small volumes are taken into account for dosimetric evaluations.

A detailed procedure for SPECT calibration is currently missing, and there is no doubt that an internationally agreed protocol would lead to further advances in this area. At present, a phantom uniformly filled with a known amount of activity or hot spheres in uniform background (as suggested by the MIRD pamphlet No. 23 [17]) are generally used in the clinical practice to calibrate the imaging system.

The obtained results confirm and extend previous studies on gamma camera calibration for quantitative SPECT imaging with ^177^Lu. Recently, two studies strictly related to this topic have been published [25,29], both testing different calibration geometries. In particular, in the first [25] the quantitative accuracy measured by using four calibration geometries (point source in air, sphere in air and in cold background, and a hot cylindrical phantom) has been compared on two different SPECT systems. The authors concluded that an accuracy to within approximately 10% can be achieved in large phantoms uniformly filled with ^177^Lu when the 208 keV photopeak is used together with suitable correction algorithms to compensate the major degrading effects. In another work [29], another four calibration geometries (hot cylindrical phantom, hot bottles in air, hot spheres in hot background with two concentration levels) have been considered. Interestingly, the authors concluded that calibration factors obtained from all tomographic acquisitions agree within 7% with each other and with the values obtained from planar scan. In the same study, the authors obtained deviations below 5% for objects larger than 100 ml in a hot background, and less than 18% for small objects, namely with a volume of about 9 ml.

Starting from these results, we have considered two calibration phantoms represented by the Hp and Js geometries. The activity concentration considered in the Hp geometry was representative of the typical activity concentration obtained in an ideal patient (70 Kg in weight) administered with 7400 MBq of ^177^Lu activity. On the other hand, the activity concentration used in the Js geometry was 30.3 MBq/ml, a situation similar to that reported in [25]. Dead time correction was measured as described in [25], and it was found to be 0.5% and 1.2% for the Hp and Js geometries, respectively. As reported in [21], the SPECT calibration factor was not greatly influenced by the choice of OSEM updates. The obtained calibration factor values for each reference geometry showed little difference between them for the fairly narrow range of OSEM update combinations considered.

One of the key issues in recovering activities in a clinical setting is the choice of the number of OSEM updates, as noise in SPECT images may be a limiting factor. As reported in MIRD pamphlet No. 26 [18], the optimal combination of subsets and iterations should be obtained considering the identical activity recovery for tumor and critical organs for the treatment (i.e. kidney in the case of neuroendocrine tumors), and those authors assumed equal volumes for both tumor and kidney. As in the clinical case the tumor volume is smaller than the kidney one, the present study focuses on small volume lesions and investigates the *COV* and *cRC* behaviors for Js and Rp phantoms (16 ml, 8 ml and 4 ml), respectively.

As for the *COV* values obtained, it is worth nothing that, contrary to expectations, *COV* values for the Js (Fig 4B) tend to decrease with increasing *EI* number up to 150 approximately, then *COV* values increase linearly with *EI* number. The reason for this trend is not yet wholly understood. However, there are several possible explanations for this result. It can be conceivably hypothesized that VOI positioning is critical and *COV* values may strongly depend on where the VOI is placed (i.e., in the middle of the phantom in the case of Js). Another possible explanation for this is that, on average, the voxel values within the sphere are gradually moving toward their “true” value, especially those near the edge of the sphere. After a number of *EI*, noise becomes dominant and *COV* values increase linearly with *EIs*. Further data collection would be needed to determine exactly how *COV* values are affected by VOI positioning, *EI* number and other minor effects.

Looking at the results shown in Fig 5 and Fig 6, the *cRC* convergence for all spheres starts at *EI* values for which the noise level is below the 17%threshold. From the results reported in Table 3, an *EI* value equal to 50 (i.e. 10*s*x5*i*) was chosen as the suitable OSEM updates. With this choice, it results that the *COV* for both Hp and Js geometries was below the established threshold. In fact, analyzing the data in Table 1 and Table 2 the Hp *COV* is 15.10%, while the Js *COV* is equal to 13.27%, representing the best compromise between the noise level for both large and small volumes. Moreover, a resolution analysis (results not shown) has been performed on a point like source filled with ^177^Lu, and the full width at half maximum (FWHM) has been evaluated as a function of 3D-OSEM updates. For *EI* = 50, the FWHM reached a plateau, while for *EI*>50 the FWHM was in convergence but the *COV* threshold of 17% is exceeded for the case of Hp geometry. Even if at an *EI* value equal to 30 the *cRC* plateau is reached (Fig 5 and Fig 6), the Js *COV* is outside of the *EI* range in which its minimum *COV* lies. In fact, the Js *COV* at *EI* value equal to 50 is lower than the one at *EI* value equal to 30 and, in the same time, the *cRC* values are in convergence. For the same reason, an *EI* value equal to 150 has been left out as the 17% threshold is exceeded for all spheres (Table 1). For the 20 and 30 subsets, the noise level reached in the reconstructed images exceeds 17% (Table 1). Moreover, for these last number of subsets the noise level for 1 and 2 iterations is lower than the established threshold (Table 1), but the *cRCs* do not reach a plateau for these combinations (data not shown in this study).

It is worth noting that our results are in agreement with previously published findings. Ilan et colleagues [9] obtained similar quantitative accuracies in small objects, using the same reference calibration geometry (Hp). Conversely, our results are higher than those reported by Sanders [21] and Uribe [29]. Anyway, a straightforward comparison between our data and the data in [21,29] is not possible because the SPECT systems, collimators, energy windows and software are different, affecting the efficiency and resolutions of the SPECT systems.

Importantly, in the present study we used a phantom made of concentric spheres to test the ability of the SPECT system to recover activity in challenging and realistic conditions. In fact, the spherical shells have the potential to reproduce a scenario of a high-uptake region surrounded by fainter circular area, which is typical of a number of clinical situations (e.g. renal medulla). As it is not possible to distinguish the spill-in and spill-out effect in this situation, the CT based contour of the inner sphere was used to estimate the reconstructed activity. Moreover, it is worth noting that the layer added by the outer sphere is about 4.8 mm, i.e. well below the system spatial resolution. As a consequence, the reconstruction and detection capability of the SPECT system are pushed to the limit.

Using the previously selected reconstruction parameters (*EI* = 50, i.e. 10*s*x5*i*), the percent difference between the reconstructed activity and the known activity inside the spherical tumor was found to be -16.4% (using the calibration factor from Hp geometry) and -24.8% (using the calibration factor from Js geometry) for the inner sphere of the insert. From these results, the Hp geometry can be considered suitable to define the SPECT calibration. Moreover, while this geometry does not include a correction for the partial volume effect, important for dosimetry of small lesions, this last correction can be accomplished by introducing the proper *cRC* as a function of the volume under investigation.

Finally, we are aware that our research may have two limitations. Firstly, activity measurements were performed using activity calibrators available in our department, with an accuracy within ±5%. It is likely that if activity is determined by a National Metrology Institute (thereby providing activity measurements with errors well below 2%), the quantitative accuracy in a clinical scenario can be greatly improved. The second limitation concerns dead-time measurements. The low activity concentration used for the assessment of dead-time through the dual source method is likely to be a possible source of inaccuracy. Notwithstanding this, the measured dead-time compares well with reported literature values [30]. Furthermore, minor uncertainties in dead-time estimates are likely to have a negligible impact on the final quantification analysis, given the relatively low activities used throughout the study.

Ultimately, we are convinced that the present study provides considerable insight into the accuracy achievable in quantitative SPECT imaging with ^177^Lu in a realistic clinical scenario. In particular our results provide further evidence for suggesting the use of a large uniform phantom as reference geometry.

## Conclusion

The use of a cylindrical homogeneous reference geometry, together with the gamma camera acquisition parameters used for SPECT image acquisition, and optimization of 3D-OSEM updates has been proved suitable for ^177^Lu SPECT activity quantification related to small volumes.

The study deeply investigates the relationship between 3D-OSEM algorithm, object size and *COV*. Considering the *cRC* together with these variables can lead to a better compromise in terms of real activity recovery, keeping the background noise as low as possible in the reconstructed SPECT images. This can be achieved by means of the homogeneous cylindrical phantom, which does not account for intrinsic corrections related to the partial volume effect with respect to a calibration sphere. This last correction can be achieved by using a proper *cRC* values calculated as a function of the volume under investigation.
